# Plasma metabolomic analysis in Thai EGFR-mutated non-small cell lung cancer patients

**DOI:** 10.1016/j.csbj.2025.10.010

**Published:** 2025-10-04

**Authors:** Lucksamon Thamlikitkul, Kwanjeera Wanichthanarak, Siriphan Manocheewa, Suphitcha Limjiasahapong, Natthaporn Phonsatta, Sujichon Thangvichien, Atikorn Panya, Yongyut Sirivatanauksorn, Naravat Poungvarin, Sakda Khoomrung

**Affiliations:** a Division of Medical Oncology, Department of Medicine, Faculty of Medicine Siriraj Hospital, Mahidol University, Bangkok, Thailand; b Siriraj Metabolomics and Phenomics Center, Faculty of Medicine Siriraj Hospital, Mahidol University, Bangkok, Thailand; c Siriraj Center of Research Excellence in Metabolomics and Systems Biology, Faculty of Medicine Siriraj Hospital, Mahidol University, Bangkok, Thailand; d Functional Ingredients and Food Biotechnology Research Unit, National Center for Genetic Engineering and Biotechnology (BIOTEC), Pathumthani, Thailand; e Department of Surgery, Faculty of Medicine Siriraj Hospital, Mahidol University, Bangkok, Thailand; f Thailand Metabolomics Association, Bangkok, Thailand; g Department of Clinical Pathology, Faculty of Medicine Siriraj Hospital, Mahidol University, Bangkok, Thailand; h Department of Biochemistry, Faculty of Medicine Siriraj Hospital, Mahidol University, Bangkok, Thailand; i Center of Excellence for Innovation in Chemistry (PERCH-CIC), Faculty of Science, Mahidol University, Bangkok, Thailand

**Keywords:** NSCLC, EGFR mutation, EGFR-TKI resistance, Plasma metabolite profiling, MS-based metabolomics

## Abstract

Lung cancer remains the leading cause of cancer-related mortality worldwide, underscoring the urgent need for non-invasive approaches to improve diagnosis, patient stratification, and therapeutic monitoring. Metabolic reprogramming driven by oncogenic alterations—particularly Epidermal Growth Factor Receptor (EGFR) mutations in non-small cell lung cancer (NSCLC)—creates distinctive plasma signatures with clinical relevance. In this study, plasma metabolomic profiling revealed that amino acid and sugar metabolism exhibited the strongest discriminatory patterns. NSCLC patients consistently showed elevated glycine and reduced tryptophan and inositol compared with healthy controls. Distinct amino acid and organic acid shifts further differentiated EGFR-mutated from wild-type NSCLC, while alterations in tryptophan, valine, and oxalic acid characterized patients with acquired resistance to EGFR tyrosine kinase inhibitors (TKIs). These findings underscore biologically relevant metabolic alterations associated with EGFR mutation and TKI resistance, supporting the potential of plasma metabolite profiles as minimally invasive indicators for molecular classification and treatment response in NSCLC.

## Highlights

• Profiles metabolic alterations in NSCLC by EGFR mutation and TKI resistance.
• Links plasma metabolites with clinical features and survival.
• Provides absolute quantification and precise metabolite identification, enhancing reproducibility.
• Reveals metabolic changes in patients with acquired EGFR TKI resistance.

## Graphical Abstract

**Figure:**
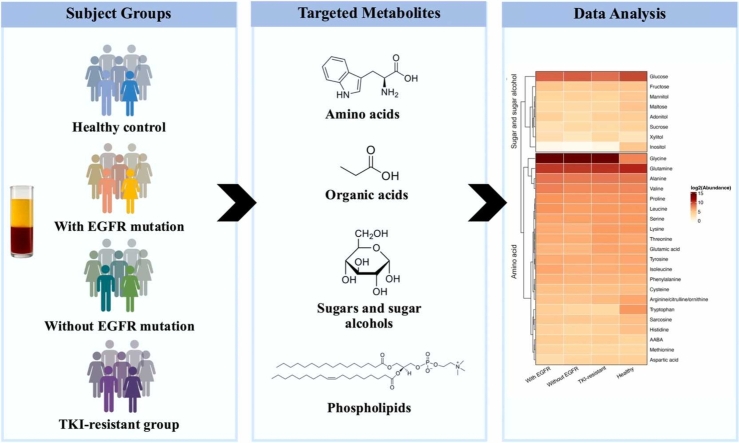

## 1 Introduction

Lung cancer is the most common cancer and the leading cause of cancer-related deaths worldwide [1]. Due to the lack of symptoms in early stages, many patients are diagnosed at advanced stages, limiting curative treatment options and contributing to high mortality. While low-dose computed tomography scan (LDCT) can detect early-stage lung cancer and reduce mortality in heavy smokers [2], it exposes patients to radiation and has a high false-positive rate, often leading to unnecessary invasive procedures. This underscores the need for a novel and less invasive screening method. Non-small cell lung cancer (NSCLC) accounts for 85 % of lung cancer cases and nearly all cases in never-smokers [3]. Treatment decisions are guided by oncogenic driver mutations, with the Epidermal Growth Factor Receptor (EGFR) mutation being the most common in non-smoking NSCLC patients—particularly among East Asian women where prevalence reaches up to 74 % [4], [5]. EGFR-mutated NSCLC responds well to EGFR tyrosine kinase inhibitor (TKI). Beyond promoting tumor growth, EGFR mutations significantly alter cancer metabolism, increasing glucose uptake, lactate production, and the pentose phosphate pathway [6], [7]. These mutations also reprogram amino acid, nucleotide, and fatty acid biosynthesis [8], resulting in distinct changes in metabolic profiles. These metabolic alterations have been well documented and present a promising avenue for developing diagnostic biomarkers [9], [10]. In Asian populations, including in Thailand, the prevalence of EGFR mutations is significantly higher than in other populations, particularly among non-smoking NSCLC patients [11]. This has important implications for treatment, as EGFR-targeted TKIs are effective in this subgroup. However, the development of acquired resistance to TKIs remains a major clinical challenge, emphasizing the need for novel biomarkers to predict treatment response and resistance mechanisms [12], [13]. Metabolomics offers a promising avenue for identifying such biomarkers [14], especially considering that cancer-associated metabolic alterations—including those driven by EGFR mutations—can vary across populations.

Liquid biopsy is a minimally invasive method commonly used to detect circulating tumor DNA (ctDNA) in the plasma, guiding treatment decisions for advanced NSCLC patients [15]. Emerging research suggests that circulating metabolites also hold promise as non-invasive biomarkers for cancer detection [16]. Although more than 150 metabolites have been linked to metabolic rewiring in lung cancer [17], variability in sample types and profiling methods has produced inconsistent results. Most prior studies have focused comparison between cancer patients and healthy controls without considering molecular subtype [17], [18]. Currently, no validated plasma biomarkers— beyond ctDNA sequencing—are available to stratify patients by EGFR mutation status or detect treatment resistance. Only one study from Korea has examined plasma metabolites in 15 NSCLC patients with and without EGFR mutation using liquid chromatography-mass spectrometry (LC-MS), identifying four potential biomarkers—linoleic acid, 5‑methyl-tetrahydrofolate, N‑succinyl‑L‑glutamate‑5-semialdehyde, and *O*-tetradecanoyl-L-carnitine — for EGFR mutation detection [19]. However, plasma metabolomic analysis in EGFR-mutated NSCLC remains limited, especially in Asian populations such as Thais, where mutation frequencies and metabolic phenotypes may differ from Western cohorts. To address this gap, we aimed to identify metabolite signatures associated with EGFR mutations and resistance to EGFR-targeted therapies. We focused on four key metabolite classes—amino acid, sugar and sugar alcohol, organic acid, and phospholipid—due to their well-established roles in cancer metabolism. These metabolite groups are closely linked to tumor biology and treatment response, making them promising candidates for disease stratification, therapeutic monitoring, and resistance prediction. Their relevance is further supported by previous studies demonstrating their utility in cancer detection and response assessment [8], [20], [21].

## 2 Experimental procedures

### 2.1 Subjects and clinical data collection

All patients had pathologically confirmed NSCLC and were treated at Siriraj Hospital, Bangkok, Thailand during January 2018 - April 2022. EGFR mutation was identified by cell-free DNA using cobas® EGFR mutation test v2 (Roche Diagnostics, Basel, Switzerland). Negative EGFR mutation results from cell-free DNA tests were confirmed with tumor tissue testing. Treatment-naïve NSCLC patients had never received any systemic treatment for lung cancer. EGFR TKI-resistant NSCLC patients had evidence of disease progression according to the Response Evaluation Criteria In Solid Tumors (RECIST) version 1.1 after receiving first- or second-generation EGFR TKI treatment. All EGFR TKI-resistant NSCLC patients had acquired the EGFR T790M mutation.

Patients’ demographic data, clinical characteristics, treatment, and treatment outcomes were collected from electronic medical records at Siriraj Hospital, Bangkok, Thailand. Progression-free survival (PFS) was defined as the duration from treatment initiation until disease progression or death. Overall survival (OS) was defined as the duration from treatment initiation until death. The study protocol was approved by the Siriraj Institutional Review Board with certificate of approval number Si 332/2022. The human studies reported in this study abide by the Declaration of Helsinki principles, the Belmont Report, CIOMS Guidelines, and the International Conference on Harmonization in Good Clinical Practice (ICH-GCP).

### 2.2 Plasma sample collection

Whole blood samples were collected from 78 participants, comprising 29 treatment-naïve NSCLC patients with EGFR mutations, 29 treatment-naïve NSCLC patients without EGFR mutations, 10 EGFR-TKI-resistant NSCLC patients, and 10 healthy individuals. Blood was drawn directly into Cell-Free DNA BCT® tubes (Streck, Nebraska, USA) to stabilize cell-free DNA. Plasma was separated from cellular components by centrifugation. For NSCLC patients, plasma was first subjected to EGFR mutation analysis. Plasma aliquots from all participants were stored at −80 °C until subsequent analysis.

### 2.3 Chemical standards and reagents

#### 2.3.1 All metabolite standards and reagents used in this study are listed in Appendix A, Appendix A

##### 2.3.1.1 Determination of amino acids by GC-MS/MS

Free amino acids were analyzed according to previous methods [22], [23]. Briefly, plasma samples (50 µL) were vortexed with 4 ml of 25 % acetonitrile in 0.1 M HCl for 2 min and sonicated at room temperature for 20 min, centrifuged at 9000 x g for 10 min. A 50 µl aliquot of the supernatant was transferred to a 2 ml GC glass vial, to which 50 µl of norleucine (200 nmol/ml) was added as an internal standard. Then, the mixture was dried at 60 °C for about 2 hr, supplemented with 50 µl dichloromethane, and dried again for 30 min. To enhance both volatility and thermal stability for GC analysis, 50 µl of the derivatizing agent N-tert-Butyldimethylsilyl-N-methyltrifluoroacetamide containing 1 % tert-Butyldimethylchlorosilane (MTBSTFA + 1 % TBDMSCl), along with 50 µl of acetonitrile, were added to the dried samples. The samples were sealed with an aluminum cap with PTFE/Red Rubber Septa and incubated at 100 °C for 4 hr in a hot-air oven and then stored at room temperature until analysis by GC-MS/MS.

The GC-MS chromatographic analysis was performed on a gas chromatograph (Agilent 7890B) coupled to a triple quadrupole mass spectrometer (Agilent 7000D) and a PAL auto sampler system (CTC Analytics AG, Switzerland). A 2 µl of sample was injected into the GC with split mode at a 1:5 split ratio at 280 °C onto a DB-5MS column (30 m, 0.25 mm i.d., Agilent J&W GC column). Helium was used as a carrier gas with a constant flow rate of 1.4 ml·min^−1^. The GC oven was programmed as follows: ramp from 130 °C to 190 °C (6 °C·min^−1^) and then to 230 °C (30 °C·min^−1^), held at 230 °C for 5 min, then ramp to 325 °C (6 °C·min^−1^), and held at 325 °C for 6 min. The transfer line, ion source, and triple quadrupole environment were set as 325 °C, 240 °C, and 180 °C, respectively. The mass spectrometer was operated in selected ion monitoring mode and used for quantitative analysis (Table S2). The calibration curves were performed using the mixture of all amino acids with different concentrations ranging from 25 to 400 nmol·ml^−1^ (Table S10). Each sample was analyzed in triplicate. Relative correction factors (RCF) were obtained as (AIS × Caa)/(Aaa × CIS) where IS = internal standard, aa = amino acid, C = concentration, A = peak area. Analysis was conducted on 60 samples daily, with QC samples (pooled) processed every 30 samples. A standard curve was generated daily using the sample matrix. The percentage relative standard deviation (% RSD) of the QC sample throughout the study was below 10 %.

### 2.4 Determination of organic acids by GC-MS/MS

Organic acids were analyzed using GC-MS/MS**.** Samples were prepared according to Evans et al. [24] with some modifications. Briefly, the sample (50 μl) was diluted with 75 µL of ultra-pure water (Type I) in a 4 ml HPLC vial. Then, 50 µL of trans-cinnamic acid (0.5 mM) was added into the vial as an internal standard, followed by 400 μl of diethyl ether to extract organic acids from the samples. The vial containing the mixture was vortexed for 2 min. After that, the mixture was centrifuged at 1400 rpm for 5 min. The 100 µL of the upper layer was collected and transferred into an autosampler crimp top vial using a positive displacement micropipette. After the organic layer was transferred, 50 µL of MTBSTFA was added as the derivatizing agent for silylation to enhance both volatility and thermal stability for GC analysis. The autosampler vial was sealed with an aluminium crimp cap with PTFE/Silicone septa. The vial was then incubated at 80 ºC for 40 min. After incubation, samples were analysed using GC-MS.

The chromatographic analysis was performed using the same system described above. Aliquots of the derivatized amino acids (1 µl) were injected using split mode at a 1:20 split ratio at 280 °C onto a DB-1MS column (30 m x 0.25μm x 0.25 mm i.d., Agilent J&W GC column). Helium was used as a carrier gas with a constant flow rate of 1.1 ml/min. The initial oven temperature was controlled at 80 °C, held for 3 min. The GC oven was programmed as follows: ramp from 80 °C to 160 °C (50 °C·min^−1^), held for 3 min and ramped from 160 °C to 340 °C (80 °C/min). The transfer line, ion source (EI), and triple quadrupole were set as 325 °C, 240 °C, and 180 °C, respectively. The mass spectrometer was operated in Multiple Reaction Monitoring (MRM) mode. MS data were acquired using MassHunter software (version 10.0, Agilent Technologies, USA) utilizing three replicates to calculate the mean and the standard error. Calibration curves were performed using the mixture of volatile (formic acid, acetic acid, propionic acid, isobutyric acid, butyric acid, isovaleric acid, valeric acid, isocaproic acid, hexanoic acid and heptanoic acid) and non-volatile (lactic acid, pyruvic acid, oxalic acid, malonic acid, methylmalonic acid, succinic acid, fumaric acid, malic acid) organic acids at different concentrations ranging from 0.78 ppm to 25 ppm (Table S10). The selected quantifier and qualifier ions are shown in Table S3). Qualitative and quantitative analyses were performed using Agilent MassHunter software (version 10.0 Agilent Technologies, USA) and exported into Microsoft Excel for further data processing. QC procedures were the same as described above, with %RSD < 10 %.

### 2.5 Determination of sugars and sugar alcohols by GC-QTOFMS

The sample preparation and analysis was adopted from Fiehn et al. [25] and Jariyasopit et al. [23] with some modifications. In brief, 50 µL of each sample was extracted with 400 µL of methanol in a 1.5 ml Eppendorf tube, vortexed at 2200 rpm for 10 min (three times), then centrifuged at 10,000 x g for 10 min (4 °C) using a centrifuge (5810 R, Eppendorf, USA). A 100 µL of supernatant was transferred into a 2 ml GC glass vial, and 20 µL of 1µmol·ml^−1^ of internal standard (myristic acid-D27) in hexane was added. The mixture was dried at 60 °C for 2 h using a vacuum concentrator (Concentrator plus, Eppendorf, USA), then supplemented with 50 µl dichloromethane and dried again for 30 min. Subsequently, a 50 µL of 40 mg·ml^−1^ of *O*-methyl hydroxylamine hydrochloride in pyridine was added to the dried sample for a methoxylation reaction. The reaction was performed at 30 °C for 90 min. For a trimethylsilylation reaction to enhance both volatility and thermal stability, 50 µL of N-trimethyl-N-methyl trifluoroacetamide containing 1 % of trimethylchlorosilane (MSTFA + 1 % TMCS) was added into the mixtures and incubated at 37 °C for 30 min. The derivatized samples were cooled at room temperature and used immediately for GC/MS analysis. The samples were analyzed by a gas chromatography-quadrupole time of flight mass spectrometer (GC/Q-TOF, GC 7890B/MSD 7250, Agilent Technologies, USA) coupled to a PAL autosampler system (CTC Analytics AG, Switzerland).

An aliquot of 2 µl was injected using split mode with an injector temperature of 250 °C and a split ratio of 10:1 onto a DB-5MS UI column (30 m, 0.25 mm i.d., Agilent Technologies, USA) in the GC/Q-TOFMS. Helium was used as a carrier gas with a constant flow rate of 1.0 ml/min. The GC oven was programmed as follows: The initial oven temperature was controlled at 60 °C, held for 1 min. Then, the temperature was ramped from 60 °C to 325 °C at the rate of 10 °C·min^−1^ and held for 10 min. The total run time was 37.5 min. The transfer line, ion source, and quadrupole were set as 325 °C, 240 °C, and 180 °C, respectively. The mass spectrometer was operated in full scan mode, ranging from *m/z* 20–1200, with a data acquisition rate of 5 Hz. MS data was acquired using MassHunter software (version 10.0, Agilent Technologies, USA), utilizing three replicates to calculate the mean and the standard error.

For targeted metabolite analysis, calibration curves were performed using the sugar mixture at different concentrations ranging from 10 to 1000 μg/ml with myristic-d_27_ acid as an internal standard (Table S10). The selected quantifier and qualifier ions are shown in Table S4. Samples were analyzed in triplicate in scan mode. Relative correction factors (RCF) were obtained as (AIS × Cs)/(As × CIS) where IS = internal standard, s = sugar, C = concentration, and A = peak area. The selected quantifier and qualifier ions are shown in Table 1. Qualitative and quantitative analyses were performed on Agilent MassHunter software (version 10.0 Agilent Technologies, USA), and exported into Microsoft Excel for further data processing. QC procedures followed the same workflow described above, with %RSD < 10 %.

**Table 1:** Clinical characteristics of NSCLC patients.

	Treatment-naïve NSCLC with EGFR mutation (n = 29)	Treatment-naïve NSCLC without EGFR mutation (n = 29)	EGFR TKI-resistant NSCLC (n = 10)
Woman – n (%)	19 (65.5)	13 (44.8)	5 (50)
Age (years) – mean (SD)	62.9 (9.1)	66.1 (11.5)	60.6 (13.3)
Smoking – n (%)	5 (17.2)	11 (37.9)	2 (20)
Adenocarcinoma – n (%)	27 (93.1)	26 (89.7)	8 (80)
EGFR mutation – n (%)			
Exon 19 deletion Exon 21 L858R	12 (41.4) 12 (41.4)	0 0	5 (50) 5 (50)
First-line treatment – n (%)			
Erlotinib Gefitinib Afatinib Osimertinib Chemotherapy No treatment	9 (31) 2 (6.9) 9 (31) 4 (13.8) 0 3 (10.3)	0 0 0 0 12 (41.4) 14 (48.3)	1 (10) 5 (50) 4 (40) 0 0 0

### 2.6 Determination of phospholipids by LC-MS/MS

Lipid extraction from plasma was adopted from Matyash et al. [26]. Briefly, 20 µL of plasma and 20 µL of 20 µM internal standard (^13^C-PC 16:0/16:0) was added to 150 µL of methanol, vortexed 30 sec, and mixed with 500 µL of methyl *tert*-butyl ether. After shaking at 2000 rpm for 30 min,125 µL of MilliQ water were added. The mixture was incubated at room temperature for 10 min and then centrifuged at 13,800 ×g for 15 min at 4 °C. The upper phase was collected, dried in a vacuum concentrator (Labconco, MO, USA), and reconstituted in 250 µL of 60:40 acetonitrile:water containing 0.1 % formic acid. Samples were vortexed for 30 sec, sonicated for 15 min, then transferred to an LC vial. Analysis was performed using a Waters Acquity I-Class UPLC coupled to a Xevo TQ-S MS/MS (ESI source). Lipids were separated on a CSH C18 column (2.1 × 100 mm, 1.7 µm; Waters, MA, USA) using solvents A (ACN/H₂O 60:40 v/v) and B (IPA/ACN 90:10 v/v), both with 10 mM ammonium formate and 0.1 % formic acid. The gradient started at 60 % A, decreased to 57 % over 2 min, changed to 50 % at 2.10 min, decreased to 46 % at 12 min, changed to 30 % at 12.10 min, decreased to 1 % by 18 min, returned to 60 % by 20 min, and held for 3 min (total run time: 23 min). Column temperature was 55 °C; flow rate: 0.3 ml·min^−1^; autosampler: 15 °C. The MS was operated in positive ionization with multiple reaction monitoring mode. Key parameters: capillary voltage 3.0 kV, source temperature 150 °C, desolvation gas (N₂) 650 L·h^−1^ at 450 °C, cone gas 150 L·h^−1^, nebulizer gas (Ar) 6.0 bar, collision gas 0.20 ml·min-1. Injection volume was 5 µL. The LC-MS/MS settings, MRM transitions and the calibration ranges are provided in Table S5. Quantification was based on matrix-matched calibration, and the target analyte peak area was normalized by the internal standard peak area. All curves had R² values > 0.99. QC samples (prepared as pooled extracts) were injected at the beginning of each run and subsequently every 20 samples. QC reproducibility was acceptable, with %RSD values below 20 % for all phospholipids. Raw data were processed using the TargetLynx (MassLynx v4.2, Waters) and exported into Microsoft Excel for further analysis.

### 2.7 Metabolite identification, data processing and statistical analysis

All sixty-nine targeted metabolites were identified at Level 1 confidence, following the standard protocol by matching retention times and mass spectrum profiles with reference standards [27]. The raw data is available at the NIH Common Fund's National Metabolomics Data Repository website, the Metabolomics Workbench, https://www.metabolomicsworkbench.org. The data can be accessed directly via the Project DOI: 10.21228/M8V84X. Twenty-one metabolites with more than 30 % missing values were excluded from the analysis. For the forty-eight metabolites included in the downstream analysis, the missing values were imputed by a random forest method. Auto scaling (i.e., each metabolite was subtracted by mean, then divided by standard deviation) and Log2 transformation of the data was performed prior to multivariate analysis and univariate analysis, respectively. To observe overall data variance, an unsupervised principal component analysis (PCA) was performed. Multiple linear models given age and gender as covariates were calculated to identify differentially changed metabolites between sample groups. Linear model fitting was performed to evaluate the associations between metabolites and clinical parameters including, OS and PFS, with β reflecting the strength and direction of association and R² indicating the goodness of fit. A false discovery rate (FDR)-adjusted p-value < 0.05 was considered to be the significance level for the comparisons of metabolites between sample groups, where significant associations between metabolites and PFS or OS were identified at the p-value < 0.05. Metabox 2.0 [28] and R version 4.3.3 were used for all the processing and analysis steps. The prediction performance of each metabolite was evaluated using logistic regression models. A 4-fold cross-validation was performed for dataset partitioning and model optimization, after which the mean values of the area under the receiver operating characteristic curve (AUC), accuracy, specificity, and sensitivity across all test sets were computed to evaluate the predictive performance. The pROC R package [29] was used for these calculations.

## 3 Results

### 3.1 Clinical characteristics of the participants

Clinical information of advanced NSCLC patients included in this study is shown in Table 1. The average age of the patients was 63.9 years, with NSCLC patients without EGFR mutations having the highest average age. The most common histology was adenocarcinoma in all groups. EGFR-mutated NSCLC patients had higher proportion of women and non-smokers compared to that of NSCLC patients without EGFR mutation. Among the healthy subjects, 9 participants (90 %) were women and the mean age (+/- standard deviation (SD)) was 44.6 (+/- 13.3) years.

### 3.2 Distinct metabolite profiling in NSCLC patients and healthy controls

**Fig. 1:**
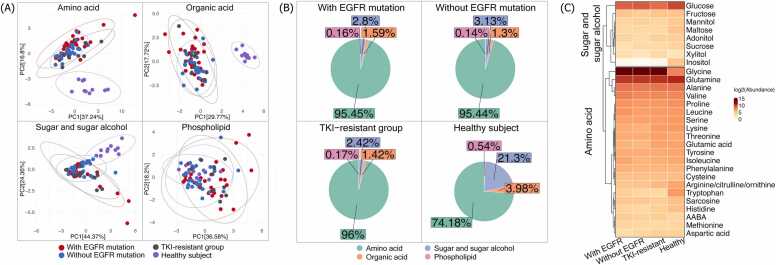
A: PCA score plots illustrated the distribution of plasma amino acid, organic acid, sugar and sugar alcohol, and phospholipid classes across four groups: NSCLC patients with EGFR mutation (N = 29, red dots), NSCLC patients without EGFR mutation (N = 29, blue dots), EGFR TKI-resistant NSCLC patients (N = 10, green dots), and healthy participants (N = 10, purple dots) B: Metabolite composition across four groups: NSCLC patients with EGFR mutation, NSCLC patients without EGFR mutation, EGFR TKI-resistant NSCLC patients, and healthy participant C: Heatmap displayed the difference in log2-transformed abundance of amino acid, and sugar and sugar alcohol classes across four groups.

This study investigated plasma metabolite profiles across four groups: 29 NSCLC patients with EGFR mutations, 29 without EGFR mutations, 10 patients resistant to EGFR-targeted therapy (TKI-resistant), and 10 healthy controls. A total of 48 metabolites were quantified, comprising 21 amino acids, 11 organic acids, 8 sugars and sugar alcohols, and 8 phospholipids. PCA based on different metabolite groups (Fig. 1A) revealed distinctions in metabolic profiles between NSCLC patients and healthy controls, particularly in the amino acid, organic acid, and sugar and sugar alcohol groups. In contrast, phospholipid profiles showed minimal variation. Metabolite composition, illustrated by pie charts (Fig. 1B), showed that amino acids accounted for over 95 % of the quantified metabolites in NSCLC patients. In comparison, healthy individuals exhibited a more diverse distribution: approximately 74 % amino acids, 21 % sugars and sugar alcohols, 4 % organic acids, and less than 1 % phospholipids. A heatmap of log2-transformed metabolite abundance (Fig. 1C) highlighted distinct metabolic patterns among the groups. Notably, lung cancer patients across all subtypes—including those with and without EGFR mutations and those with TKI resistance—consistently exhibited elevated levels of glycine and reduced levels of tryptophan and inositol compared to healthy controls.

**Fig. 2:**
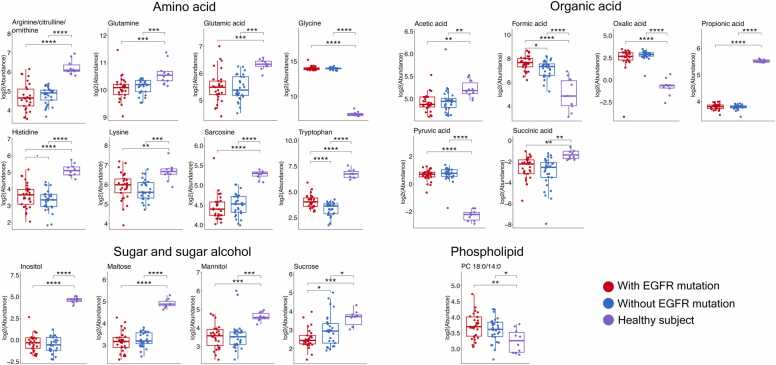
Significantly altered plasma metabolites between NSCLC patients [(with (N = 29) and without EGFR mutation (N = 29)] and healthy subjects (N = 10). The box and whisker plots demonstrated the log2-levels of the top candidates from amino acid, organic acid, sugar and sugar alcohol, and phospholipid classes, which were significantly different between NSCLC patients and healthy subjects. Red, NSCLC patients with EGFR mutation; Blue, NSCLC patients without EGFR mutation; Purple, healthy subjects; • adjusted p < 0.1; *, adjusted p < 0.05; **, adjusted p < 0.01; ***, adjusted p < 0.001; ****, adjusted p < 0.0001. The summary of all quantified metabolites and multiple regression analysis are listed in the Table S8.

Next, we analyzed metabolites that significantly differed between NSCLC patients (with and without EGFR mutations) and healthy controls (Fig. 2). Regardless of EGFR mutation status, NSCLC patients showed significant differences in amino acid levels compared to healthy individuals. Glycine was strongly elevated in NSCLC patients (Log2FC > 6.0 and p < 0.0001, Table S8), while several amino acids—including histidine, tryptophan, sarcosine, arginine/citrulline/ornithine (all p < 0.0001), glutamine, glutamic acid (both p < 0.001), and lysine (p < 0.01) were decreased.

Among organic acids, propionic acid (p < 0.0001), acetic acid, and succinic acid (both p < 0.01) were reduced, whereas pyruvic, formic, and oxalic acids were elevated (all p < 0.0001). Sugars and sugar alcohols also differed: inositol and maltose (both p < 0.0001), sucrose (p < 0.05), and mannitol (p < 0.001) were consistently lower in NSCLC patients. Phospholipid analysis revealed a modest but significant increase in phosphatidylcholine (PC 18:0/14:0) (p < 0.05) in lung cancer patients. Complete data are provided in Table S8. We further evaluated the performance of plasma metabolites to predict NSCLC patients from healthy subjects. Top ten plasma metabolites having highest accuracy for detecting NSCLC patients were listed in Table S6.

### 3.3 Metabolite profiling in NSCLC patients stratified by EGFR mutation and treatment status

**Fig. 3:**
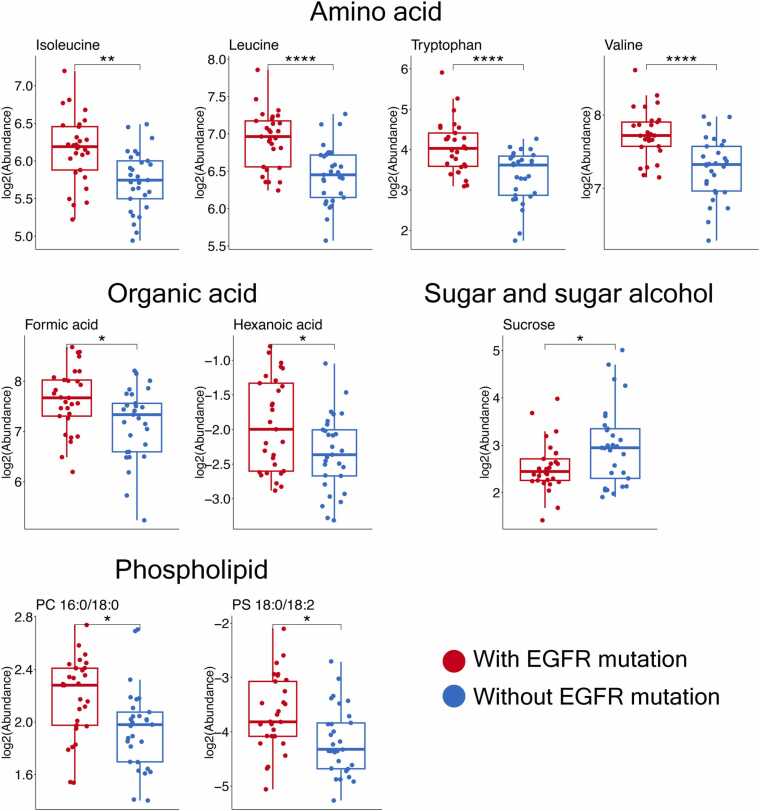
Significantly altered plasma metabolites between NSCLC patients with (N = 29) and without EGFR mutation (N = 29). The box and whisker plots demonstrated the log2-levels of amino acid, organic acid, sugar and sugar alcohol, and phospholipid classes, which were significantly different between NSCLC patients with and without EGFR mutation. Red, NSCLC patients with EGFR mutation; Blue, NSCLC patients without EGFR mutation; adjusted p < 0.1; *, adjusted p < 0.05; **, adjusted p < 0.01; ***, adjusted p < 0.001; ****, adjusted p < 0.0001. The summary of all quantified metabolites and multiple regression analysis are listed in the Table S8.

We observed significant differences in plasma metabolite profiles between NSCLC patients with and without EGFR mutations (Fig. 3). Four amino acids—tryptophan, valine, leucine (all p < 0.0001), and isoleucine (p < 0.01)—along with two organic acids (formic acid and hexanoic acid, both p < 0.05) and two phospholipids (PS 18:0/18:2 and PC 16:0/18:0, both p < 0.05), were significantly elevated in patients with EGFR mutations despite their low magnitudes of Log2FC values (< 1.0, Table S8). In contrast, sucrose was the only metabolite found at higher levels in patients without the mutation (p < 0.05). Amino acids thus represented most statistically significant metabolites and ranked among the top ten plasma metabolites with the highest predictive accuracy for distinguishing between EGFR mutation statuses (Table S7). Valine and leucine showed comparable prediction performance, although valine had higher accuracy and sensitivity than leucine (Table S7). Specifically, valine achieved an AUC > 0.7 with strong accuracy and sensitivity, supporting its robustness as a classifier of EGFR mutation status.

**Fig. 4:**
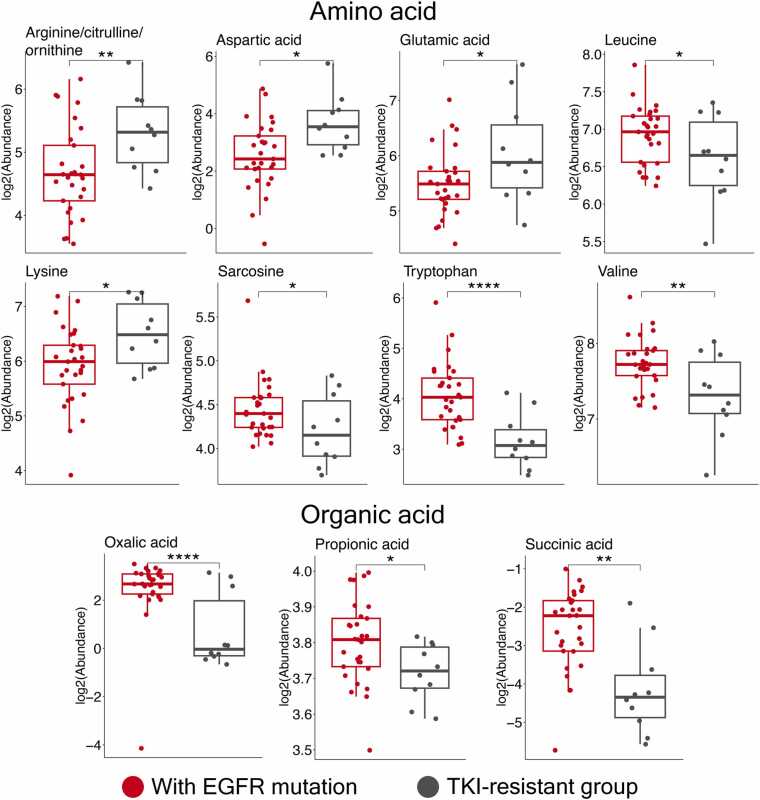
Significantly altered plasma metabolites between treatment-naïve NSCLC patients with EGFR mutation (N = 29) and EGFR TKI-resistant NSCLC patients (N = 10). The box and whisker plots demonstrated the levels of metabolites from amino acid and organic acid classes, which were significantly different between EGFR-mutated NSCLC patients before receiving EGFR TKI treatment and after developing resistance to EGFR TKI. Red, treatment-naïve NSCLC patients with EGFR mutation; Green, EGFR TKI-resistant NSCLC patients; *, adjusted p < 0.05; **, adjusted p < 0.01; ***, adjusted p < 0.001; ****, adjusted p < 0.0001. The summary of all quantified metabolites and multiple regression analysis are listed in the Table S8.

Although advanced NSCLC patients harboring sensitizing EGFR mutations usually respond well to EGFR TKI during the first 1–1.5 year of treatment [30], most patients eventually develop resistance, leading to tumor regrowth or metastasis. Hence, we focused on distinguishing between NSCLC patients with EGFR mutations who were treatment-naïve and those who had developed resistance to EGFR TKI. In patients with acquired EGFR TKI resistance, we observed pronounced alterations in plasma metabolites (Fig. 4). Several amino acids were reduced, including tryptophan (p < 0.0001), valine (p < 0.01), leucine (p < 0.01), sarcosine (p < 0.05). Organic acids such as oxalic acid (p < 0.0001), propionic acid (p < 0.05), and succinic acid (p < 0.01) were also decreased. Although changes in sugars, sugar alcohols, and phospholipids did not reach statistical significance, their levels tended to be higher in EGFR TKI-resistant patients (Table S8). Conversely, resistant patients showed modest increases in arginine/citrulline/ornithine (p < 0.01), aspartic acid, lysine, and glutamic acid (all p < 0.05), compared to treatment-naïve EGFR- mutated NSCLC patients, although the effect sizes were small (Log2FC < 1.0, Table S8).

We next assessed the predictive performance of plasma metabolites in distinguishing between these two patient groups. Among the top ten plasma discriminatory metabolites, tryptophan and succinic acid showed the highest classification accuracy (Table S9). Tryptophan, in particular, achieved strong AUC, accuracy, and sensitivity values (>0.8), although its specificity was modest (0.625) (Table S9). This suggests that tryptophan is effective at identifying true positives but may introduce more false positives. Importantly, tryptophan was only the metabolite that consistently differed across all group comparisons: healthy controls vs. NSCLC patients, EGFR-mutant vs. wild-type NSCLC, and treatment-naïve vs. TKI-resistant EGFR-mutant NSCLC. In every case, healthy individuals exhibited significantly higher tryptophan levels than NSCLC patient groups.

### 3.4 Correlations between plasma metabolite profiles and patient’s clinical characteristics

**Fig. 5:**
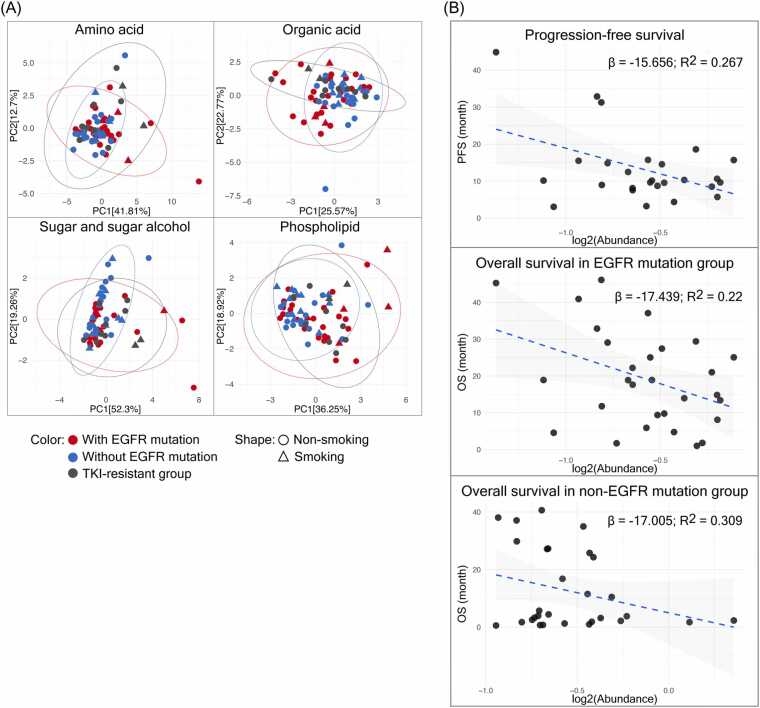
A: Principal component analysis of metabolite profiles—amino acid, organic acid, sugar and sugar alcohol, and phospholipid classes—stratified by smoking status. B: Associations between plasma isobutyric acid levels and clinical outcomes: progression-free survival (PFS) in NSCLC patients treated with EGFR TKIs (N = 10 left), overall survival (OS) in NSCLC patients with EGFR mutations (N = 29, middle), and OS in those without EGFR mutations (N = 29, right). β represents the regression slope, R² indicates the goodness of fit and the shaded area depicts the 95 % confidence interval.

We examined the associations between plasma metabolites and key clinical parameters in NSCLC patients. Specifically, we examined differences based on smoking status across all patient groups, correlations with OS in EGFR-mutant and wild-type cases, and the links to PFS in patients receiving EGFR TKI therapy. Smoking influences lung cancer risk and mutation patterns, while EGFR mutations are more frequent in non-smokers, with prior studies also linking smoking status to serum metabolite profiles [31]. To explore this potential relationship in the context of our study, we performed PCA of plasma metabolites from all NSCLC patient groups, excluding the healthy control group. As illustrated in Fig. 5A, plasma metabolite profiles did not clearly differ between patients categorized by smoking status. Metabolite patterns, including amino acids, organic acids, sugars and sugar alcohols, and phospholipids, appeared similar across both smoker and non-smoker groups. In addition, non-smokers were predominant in the EGFR mutation group, whereas in the EGFR wild-type group, the proportions of smokers and non-smokers were relatively similar (60:40) (Fig. 5A and Table 1).

For advanced-stage NSCLC patients harboring the EGFR mutation, EGFR TKI is the standard first-line treatment, known to enhance treatment responses and PFS of the patients. We investigated whether plasma metabolite levels correlate with the PFS among patients who received EGFR TKI, as well as with OS across EGFR-mutant and wild-type patient groups. Isobutyric acid was identified as a significant metabolite (Fig. 5B) associated with both PFS and OS. Higher isobutyric acid levels correlated with shorter PFS (p = 0.012, R² = 0.267) and decreased OS in both EGFR-mutant (p = 0.028, R² = 0.22) and EGFR wild-type patients (p = 0.045, R² = 0.309).

## 4 Discussion

### 4.1 Changes in amino acid metabolism in EGFR-mutant NSCLC

Consistent with previous studies [17], [18], NSCLC patients exhibited broad disturbances in amino acid metabolism. Several amino acids—including tryptophan, sarcosine, arginine/citrulline/ornithine, histidine, glutamine, and lysine—were reduced in plasma, potentially reflecting increased tumor utilization. Glutamine depletion supports the concept of “glutamine addiction” in cancer cells, as it serves as a carbon source for nucleotide synthesis and anaplerosis [32], [33]. Likewise, reduced tryptophan may reflect overexpression of indoleamine-2,3-dioxygenase in the tumor microenvironment, driving metabolism toward kynurenine production and immune tolerance [34]. Because of the age differences between the control and NSCLC groups, the observed decline in tryptophan in this study may partly reflect age effects. This consideration is important, as previous studies have shown that plasma glycine and inositol levels remain relatively stable with age in adults, whereas tryptophan levels consistently decline [35], [36]. However, since the NSCLC patient subgroups in our study did not differ significantly in age, the observed differences in tryptophan are more likely attributable to disease- or mutation-specific states.

Among NSCLC patients, EGFR-mutated cases exhibited distinct profiles, including elevated branched-chain amino acids (BCAAs: valine, leucine, and isoleucine), and shifts in organic acid metabolism. These findings are consistent with mechanistic evidence that EGFR mutations promote amino acid synthesis and BCAA dependence to sustain proliferation [8]. Compared with the only prior plasma metabolomics study, performed in Korea [19], our work provides novelty in three respects: i) a larger cohort (78 vs. 15 participants), ii) use of targeted GC–MS and LC–MS/MS for absolute quantification and iii) stratification by both EGFR status and TKI resistance. These design features and quantitative results enhance reproducibility and translational relevance [37].

### 4.2 Metabolic markers of TKI resistance

A notable observation was the metabolomic shift accompanying acquired EGFR TKI resistance. Resistant patients showed decreased amino acids (tryptophan, valine, leucine, sarcosine) and organic acids (oxalic acid, succinic acid, propionic acid), but increased aspartic acid, lysine, arginine/citrulline/ornithine, and glutamic acid. These adaptations may serve as bypass pathways, compensating for drug-induced metabolic bottlenecks and sustaining tumor growth. These alterations suggest compensatory metabolic rewiring that enables tumor cells to adapt and survive under TKI pressure, consistent with recent evidence of metabolic plasticity during acquired resistance [38]. In our cohort, tryptophan and oxalic acid emerged as strong predictors of acquired resistance, with the caveat that tryptophan is age-associated. While these markers are study-specific, they point toward broader metabolic vulnerabilities that may be therapeutically exploitable. Consistent with this concept, recent work has demonstrated metabolism-directed strategies to overcome EGFR-TKI resistance, including inhibition of BCAT1/BCAA metabolism and disruption of kynurenine–AHR signaling in the tumor microenvironment [39], [40].

### 4.3 Effects of ethnicity, population and lifestyle behaviors on the metabolome

Genetic and dietary factors, particularly those unique to the Thai population, may influence baseline metabolic profiles. Traditional Thai diets, rich in rice, seafood, fruits, and vegetables, could contribute various amino acids, sugars, organic acids, and fatty acids that shape metabolic signatures [41], [42], [43]. Against this background, our study provides the first evidence of significant plasma metabolite alterations among Thai NSCLC patients compared to healthy controls and across subgroups defined by EGFR mutation and resistance status. While many metabolite changes were concordant with earlier reports, some discrepancies highlight the importance of population-specific analyses. For example, glycine was elevated in our Thai cohort and in a Chinese cohort [44] but often reduced in Western studies [18]. This variability in lung-cancer metabolomics likely reflects tumor-intrinsic heterogeneity and host context. Large cohort data show subgroup-dependent pathway signals (e.g., lipid vs amino-acid pathways by smoking status), while reviews highlight that diet and lifestyle further shape systemic metabolite profiles [17], [45], [46]. Glycine metabolism is context-dependent, supporting proliferation in some settings and secretion in others, underscoring the need for standardized, multi-ethnic studies.

We also observed no significant differences in plasma metabolite profiles between smokers and non-smokers with NSCLC, suggesting that EGFR mutation status exerts a stronger influence on metabolic phenotypes than smoking history. A previous study, however, demonstrated that smoking can markedly alter systemic metabolism of organic acids and amino acids [47]. In parallel, lung cancer in never-smokers has emerged as a distinct clinical and biological entity, more common in women and Asian populations [48], and strongly associated with oncogenic drivers such as EGFR mutations [49]. Notably, our cohort was composed predominantly of non-smokers with adenocarcinoma, consistent with the higher frequency of EGFR mutations in Asian populations. These observations underscore the need to interpret plasma metabolomic signatures within the dual context of smoking history and molecular genotype, while recognizing that in our cohort, tumor genomic status appeared to dominate metabolic phenotypes.

Exploratory survival analysis revealed a trend toward worse PFS and OS with elevated plasma isobutyric acid in NSCLC patients, although the association did not reach strong statistical significance. Isobutyric acid originates from gut microbial valine fermentation [50]. It has been linked to immune evasion in colorectal cancer [50], while a Chinese study reported inhibitory effects on lung cancer cells and association with therapy response [51]. However, that cohort differed markedly from ours: half were smokers, most had squamous or small-cell carcinoma, and most received chemotherapy, whereas our patients were predominantly non-smokers with adenocarcinoma (∼ 90 %). These differences highlight how ethnicity, lifestyle, tumor subtype, and treatment history shape the metabolome and its prognostic associations.

### 4.4 Clinical and translational implications

Despite growing interest, clinical evidence supporting routine blood-based biomarkers for lung cancer remains limited. ctDNA has been studied extensively, but its sensitivity and specificity can be constrained, particularly in early disease. Plasma metabolite profiling offers a complementary modality: targeted metabolite panels could augment ctDNA for early detection in high-risk individuals, support minimal residual disease monitoring after curative surgery, and provide pharmacodynamic readouts of therapy response or resistance in advanced disease. From a practical perspective, plasma metabolomics could be integrated into clinical workflows, as sample collection is minimally invasive and mass spectrometry platforms are increasingly available in hospital laboratories. Compared with ctDNA, metabolite profiling may offer faster turnaround and lower cost, though ctDNA currently provides higher specificity for mutation detection. Importantly, individual plasma metabolites are unlikely to serve as stand-alone biomarkers given their susceptibility to dietary, metabolic, and organ-function variability; instead, targeted metabolite panels integrated with ctDNA and clinical factors are more likely to yield robust diagnostic and monitoring performance.

Our findings nominate a focused set of metabolites that discriminate NSCLC from healthy controls, differentiate EGFR mutation status, and predict resistance to EGFR TKIs. Rather than serving as isolated markers, these metabolites should be prioritized for development into a targeted panel integrated with ctDNA and clinical factors. Such a multimodal strategy has the potential to deliver a more robust, scalable, and clinically actionable approach to lung cancer screening, therapeutic monitoring, and resistance prediction.

### 4.5 Study limitations and future directions

This study has several strengths, including targeted metabolomics with absolute quantification, subgroup stratification by EGFR mutation and resistance, and correlation with clinical outcomes, thereby addressing reproducibility concerns noted in prior studies [52]. Nonetheless, several limitations should be acknowledged. First, the GC–MS derivatization method converted both arginine and citrulline to ornithine, preventing separate quantification [23], [53]. Future work should incorporate complementary approaches such as LC–MS/MS without derivatization.

The relatively small sample size, cross-sectional design, and absence of an external validation cohort limit causal inference, generalizability, and temporal assessment of disease progression or resistance. Overfitting is a further concern, reflected in modest specificity values of the TKI resistance models, and the subgroup with acquired resistance was particularly small (n = 10). The healthy control cohort was also minimally phenotyped, with limited demographic, clinical, and lifestyle data, introducing potential residual confounding factors, including age as mentioned above. Age differences between controls and NSCLC patients may have influenced the observed decline in tryptophan, as prior studies indicate that tryptophan levels consistently decrease with age, whereas glycine and inositol remain relatively stable.

Finally, while metabolite changes were interpreted in the context of oncogenic pathways, the lack of integrated genomic or transcriptomic data restricted mechanistic depth. Future research should validate these findings in larger, multi-ethnic, longitudinal cohorts; apply standardized protocols (e.g., fasting samples, detailed dietary and lifestyle data); and develop parsimonious metabolite panels optimized for cost, reproducibility, and clinical translation.

In summary, this study advances understanding of NSCLC metabolic heterogeneity by linking plasma metabolite profiles to EGFR mutation, TKI resistance, and Thai-specific patient context. These insights provide a foundation for targeted panel development, integration of metabolomics with ctDNA and genomic data, and the design of prospective trials. Ultimately, population-informed metabolomics can move beyond descriptive profiling to enable precision strategies for lung cancer detection, monitoring, and treatment.

## 5 Conclusions

Our targeted plasma metabolomics analysis yielded three main insights into NSCLC. First, stable metabolites, particularly glycine, consistently distinguished patients from controls. Second, EGFR mutation and resistance were linked to distinct metabolic profiles: EGFR-mutated tumors showed BCAA –dependent phenotype, while resistance to EGFR TKIs involved rewiring of amino acid and organic acid pathways, with tryptophan and oxalic acid showing predictive potential. Third, Thai-specific patterns, including elevated glycine and prognostic associations with isobutyric acid, underscored the influence of ethnic and dietary context. Collectively, these findings advance understanding of NSCLC metabolic heterogeneity, highlight clinically relevant features for validation in larger multi-ethnic cohorts, and point toward more precise strategies for patient stratification, monitoring, and resistance prediction—bridging metabolomics with precision oncology.

## Author contributions

The manuscript was written through contributions of all authors. All authors have given approval to the final version of the manuscript. Concept and design: **Thamlikitkul** and **Khoomrung** Acquisition of data: **Thamlikitkul, Manocheewa, Limjiasahapong, Phonsatta, Thangvichien, Panya, Poungvarin**, and **Khoomrung**. Analysis and interpretation of data: **Thamlikitkul, Wanichthanarak, Manocheewa, Limjiasahapong, Phonsatta, Panya**, and **Khoomrung**. Drafting of the manuscript: **Thamlikitkul** and **Khoomrung**. Critical revision of the paper for important intellectual content: **Thamlikitkul, Wanichthanarak, Manocheewa, Limjiasahapong, Phonsatta, Thangvichien, Panya, Sirivatanauksorn, Poungvarin**, and **Khoomrung**. Statistical analysis: **Thamlikitkul, Wanichthanarak**, and **Khoomrung**. Figure preparation: **Wanichthanarak**, and **Khoomrung**. Provision of study materials or patients: **Thamlikitkul** and **Poungvarin**. Funding and resources: **Thamlikitkul, Sirivatanauksorn**, and **Khoomrung**. Administrative, technical, or logistic support: **Thamlikitkul, Sirivatanauksorn, Poungvarin**, and **Khoomrung**.

## CRediT authorship contribution statement

**Atikorn Panya:** Writing – review & editing, Resources, Methodology, Investigation, Funding acquisition, Formal analysis, Data curation. **Yongyut Sirivatanauksorn:** Writing – review & editing, Supervision, Resources, Funding acquisition, Conceptualization. **Naravat Poungvarint:** Writing – review & editing, Supervision, Resources, Formal analysis, Conceptualization. **Siriphan Manocheewa:** Writing – review & editing, Methodology, Formal analysis, Data curation. **Suphitcha Limjiasahapong:** Writing – review & editing, Methodology, Formal analysis, Data curation. **Natthaporn Phonsatta:** Writing – review & editing, Methodology, Investigation, Formal analysis, Data curation. **Sujichon Thangvichien:** Writing – review & editing, Methodology, Investigation, Formal analysis, Data curation. **Sakda Khoomrung:** Writing – review & editing, Writing – original draft, Supervision, Resources, Project administration, Investigation, Funding acquisition, Formal analysis, Data curation, Conceptualization. **Lucksamon Thamlikitkul:** Writing – review & editing, Writing – original draft, Visualization, Resources, Project administration, Investigation, Funding acquisition, Formal analysis, Conceptualization. **Kwanjeera Wanichthanarak:** Writing – review & editing, Visualization, Validation, Methodology, Investigation, Formal analysis, Data curation.

## Funding sources

This study was funded by The Foundation For Cancer Care Siriraj Hospital (Grant number R016541068) to LT. This research received funding from the National Science, Research, and Innovation Fund (NSRF) via the Program Management Unit for Human Resources & Institutional Development, Research, and Innovation, Grant no. B36G660007 to SK. Genomics Thailand, Health Systems Research Institute (HSRI), Thailand, partially supported this study (grant number 68-129) to LT and SK. Additional support was provided by the Center of Excellence for Innovation in Chemistry (PERCH-CIC), the Ministry of Higher Education, Science, Research, and Innovation, Thailand; the Research Excellence Development (RED) Program, Faculty of Medicine Siriraj Hospital, Mahidol University; and the Siriraj Research Development Fund (Grant No. R016737002). The funders had no role in the design, conduct, analysis, interpretation, or publication of this study.

## Declaration of Competing Interest

The authors declare that there is no conflict of interest.

## Data Availability

This article contains supporting information in supplementary table and figures. The data will be made available on request.

## Footnotes

Appendix A Supplementary data associated with this article can be found in the online version at doi:10.1016/j.csbj.2025.10.010.
