# Supplementary material for: Plasma metabolomic analysis in Thai EGFR-mutated non-small cell lung cancer patients
Source: Comput Struct Biotechnol J. 2025 Oct 4;27:4321–31. doi: 10.1016/j.csbj.2025.10.010 (PMC12546685; doi:10.1016/j.csbj.2025.10.010)
Supplement: Supplementary file 1 — Supplementary material [file mmc1.docx]

**Supplementary information**

**Plasma Metabolomic Analysis in Thai EGFR-mutated Non-Small Cell Lung Cancer Patients**

Lucksamon Thamlikitkul^1^, Kwanjeera Wanichthanarak^2,3^, Siriphan Manocheewa^2^, Suphitcha Limjiasahapong^2^, Natthaporn Phonsatta^4^, Sujichon Thangvichien^4^, Atikorn Panya^4^, Yongyut Sirivatanauksorn^2,5,6^, Naravat Poungvarin^7^, Sakda Khoomrung^2,3,6 ,8, 9,*^

^1^Division of Medical Oncology, Department of Medicine, Faculty of Medicine Siriraj Hospital, Mahidol University, Bangkok, Thailand

^2^Siriraj Metabolomics and Phenomics Center, Faculty of Medicine Siriraj Hospital, Mahidol University, Bangkok, Thailand

^3^Siriraj Center of Research Excellence in Metabolomics and Systems Biology, Faculty of Medicine Siriraj Hospital, Mahidol University, Bangkok, Thailand

^4^Functional Ingredients and Food Biotechnology Research Unit, National Center for Genetic Engineering and Biotechnology (BIOTEC), Pathumthani, Thailand

^5^Department of Surgery, Faculty of Medicine Siriraj Hospital, Mahidol University, Bangkok, Thailand

^6^Thailand Metabolomics Association, Bangkok, Thailand

^7^Department of Clinical Pathology, Faculty of Medicine Siriraj Hospital, Mahidol University, Bangkok, Thailand

^8^Department of Biochemistry, Faculty of Medicine Siriraj Hospital, Mahidol University, Bangkok, Thailand

^9^Center of Excellence for Innovation in Chemistry (PERCH-CIC), Faculty of Science, Mahidol University, Bangkok, Thailand.

*Sakda Khoomrung: <https://orcid.org/0000-0001-9461-8597>

Sakda Khoomrung, Email: [sakda.kho@mahidol.edu](mailto:sakda.kho@mahidol.edu)

**KEYWORDS** lung cancer, EGFR mutation, metabolomics, plasma metabolite, biomarker

Table S1 Chemical standards and reagents

| Order | Name | Class* | Purity | From |
| --- | --- | --- | --- | --- |
| 1 | Alanine | Amino acid | >99% | Tokyo Chemical Industry Co., Ltd. (Tokyo, Japan) |
| 2 | Glycine | Amino acid | >99% | Tokyo Chemical Industry Co., Ltd. (Tokyo, Japan) |
| 3 | Sarcosine | Amino acid | >98% | Tokyo Chemical Industry Co., Ltd. (Tokyo, Japan) |
| 4 | α-Aminobutyric acid | Amino acid | >98% | Thermo Fisher Scientific Inc. (Waltham, USA) |
| 5 | Valine | Amino acid | >98% | Tokyo Chemical Industry Co., Ltd. (Tokyo, Japan) |
| 6 | Leucine | Amino acid | >99% | Tokyo Chemical Industry Co., Ltd. (Tokyo, Japan) |
| 7 | Isoleucine | Amino acid | >98% | Tokyo Chemical Industry Co., Ltd. (Tokyo, Japan) |
| 8 | Proline | Amino acid | >99% | Tokyo Chemical Industry Co., Ltd. (Tokyo, Japan) |
| 9 | Methionine | Amino acid | >99% | Tokyo Chemical Industry Co., Ltd. (Tokyo, Japan) |
| 10 | Serine | Amino acid | >99% | Tokyo Chemical Industry Co., Ltd. (Tokyo, Japan) |
| 11 | Threonine | Amino acid | >99% | Tokyo Chemical Industry Co., Ltd. (Tokyo, Japan) |
| 12 | Phenylalanine | Amino acid | >98% | Tokyo Chemical Industry Co., Ltd. (Tokyo, Japan) |
| 13 | Aspartic acid | Amino acid | >99% | Tokyo Chemical Industry Co., Ltd. (Tokyo, Japan) |
| 14 | Cysteine | Amino acid | ≥98% | Sigma-Aldrich Co., (St. Louis, USA) |
| 15 | Glutamic acid | Amino acid | >99% | Tokyo Chemical Industry Co., Ltd. (Tokyo, Japan) |
| 16 | Ornithine | Amino acid | >98% | Tokyo Chemical Industry Co., Ltd. (Tokyo, Japan) |
| 17 | Asparagine | Amino acid | >99% | Tokyo Chemical Industry Co., Ltd. (Tokyo, Japan) |
| 18 | Lysine | Amino acid | >97% | Tokyo Chemical Industry Co., Ltd. (Tokyo, Japan) |
| 19 | Glutamine | Amino acid | >99% | Tokyo Chemical Industry Co., Ltd. (Tokyo, Japan) |
| 20 | Arginine | Amino acid | >99% | Tokyo Chemical Industry Co., Ltd. (Tokyo, Japan) |
| 21 | Histidine | Amino acid | >99% | Tokyo Chemical Industry Co., Ltd. (Tokyo, Japan) |
| 22 | Tyrosine | Amino acid | >98.5% | Tokyo Chemical Industry Co., Ltd. (Tokyo, Japan) |
| 23 | Tryptophan | Amino acid | >98.5% | Tokyo Chemical Industry Co., Ltd. (Tokyo, Japan) |
| 24 | Norleucine | Amino acid (IS) | 99% | Thermo Fisher Scientific Inc. (Waltham, USA) |
| 25 | Formic acid | Organic acid | >98.0% | Tokyo Chemical Industry Co., Ltd. (Tokyo, Japan) |
| 26 | Acetic acid | Organic acid | >99.5% | Tokyo Chemical Industry Co., Ltd. (Tokyo, Japan) |
| 27 | Propionic acid | Organic acid | >99.0% | Tokyo Chemical Industry Co., Ltd. (Tokyo, Japan) |
| 28 | Isobutyric acid | Organic acid | >99.0% | Tokyo Chemical Industry Co., Ltd. (Tokyo, Japan) |
| 29 | Butyric acid | Organic acid | >99.0% | Tokyo Chemical Industry Co., Ltd. (Tokyo, Japan) |
| 30 | Isovaleric acid | Organic acid | >99.0% | Tokyo Chemical Industry Co., Ltd. (Tokyo, Japan) |
| 31 | Valeric acid | Organic acid | >98.0% | Tokyo Chemical Industry Co., Ltd. (Tokyo, Japan) |
| 32 | Isocaproic acid | Organic acid | >97.0% | Tokyo Chemical Industry Co., Ltd. (Tokyo, Japan) |
| 33 | Hexanoic acid | Organic acid | >98.0% | Tokyo Chemical Industry Co., Ltd. (Tokyo, Japan) |
| 34 | Heptanoic acid | Organic acid | ≥99% | Sigma-Aldrich Co., (St. Louis, USA) |
| 35 | Lactic acid | Organic acid | ~90% | Fluka Chemie (Buchs, Switzerland) |
| 36 | Oxalic acid | Organic acid | ≥99% | Fluka Chemie (Buchs, Switzerland) |
| 37 | Oxaloacetic acid | Organic acid | ≥99% | Fluka Chemie (Buchs, Switzerland) |
| 38 | Malonic acid | Organic acid | >99% | Merck Schuchardt OHG ([Hohenbrunn, Germany)](https://www.chemeurope.com/en/companies/11006/merck-schuchardt-ohg.html) |
| 39 | Methylmalonic acid | Organic acid | >98.0% | Tokyo Chemical Industry Co., Ltd. (Tokyo, Japan) |
| 40 | Succinic acid | Organic acid | 99.5% | Sigma-Aldrich Co., (St. Louis, USA) |
| 41 | Fumaric acid | Organic acid | >99.0% | Tokyo Chemical Industry Co., Ltd. (Tokyo, Japan) |
| 42 | Pyruvic acid | Organic acid | >97.0% | Tokyo Chemical Industry Co., Ltd. (Tokyo, Japan) |
| 43 | Malic acid | Organic acid | >99.0% | Tokyo Chemical Industry Co., Ltd. (Tokyo, Japan) |
| 44 | Trans-cinnamic acid | Organic acid (IS) | ≥99% | Sigma-Aldrich Co., (St. Louis, USA) |
| 45 | Erythritol | Sugar and sugar alcohol | ≥95% | Sigma-Aldrich Co., (St. Louis, USA) |
| 46 | Xylose | Sugar and sugar alcohol | ≥99% | Sigma-Aldrich Co., (St. Louis, USA) |
| 47 | Arabinose | Sugar and sugar alcohol | ≥98% | Sigma-Aldrich Co., (St. Louis, USA) |
| 48 | Ribose | Sugar and sugar alcohol | ≥99% | Sigma-Aldrich Co., (St. Louis, USA) |
| 49 | Xylitol | Sugar and sugar alcohol | ≥99% | Sigma-Aldrich Co., (St. Louis, USA) |
| 50 | Arabitol | Sugar and sugar alcohol | ≥99% | Sigma-Aldrich Co., (St. Louis, USA) |
| 51 | Adonitol | Sugar and sugar alcohol | ≥99% | Sigma-Aldrich Co., (St. Louis, USA) |
| 52 | Fructose | Sugar and sugar alcohol | ≥99% | Sigma-Aldrich Co., (St. Louis, USA) |
| 53 | Mannose | Sugar and sugar alcohol | ≥99% | Sigma-Aldrich Co., (St. Louis, USA) |
| 54 | Galactose | Sugar and sugar alcohol | ≥99% | Sigma-Aldrich Co., (St. Louis, USA) |
| 55 | Glucose | Sugar and sugar alcohol | ≥99.5% | Sigma-Aldrich Co., (St. Louis, USA) |
| 56 | Mannitol | Sugar and sugar alcohol | ≥98% | Sigma-Aldrich Co., (St. Louis, USA) |
| 57 | Sorbitol | Sugar and sugar alcohol | ≥98% | Sigma-Aldrich Co., (St. Louis, USA) |
| 58 | Dulcitol | Sugar and sugar alcohol | ≥99% | Sigma-Aldrich Co., (St. Louis, USA) |
| 59 | Inositol | Sugar and sugar alcohol | ≥99% | Sigma-Aldrich Co., (St. Louis, USA) |
| 60 | Sucrose | Sugar and sugar alcohol | ≥99.5% | Sigma-Aldrich Co., (St. Louis, USA) |
| 61 | Lactose | Sugar and sugar alcohol | ≥99% | Sigma-Aldrich Co., (St. Louis, USA) |
| 62 | Maltose | Sugar and sugar alcohol | ≥99% | Sigma-Aldrich Co., (St. Louis, USA) |
| 63 | Maltitol | Sugar and sugar alcohol | ≥98% | Sigma-Aldrich Co., (St. Louis, USA) |
| 64 | Myristic acid D-27 | IS | 98% | Cambridge Isotope Laboratories, Inc. (Massachusetts, USA) |
| 65 | PI 18:1/18:1 | Phospholipids | >99% | Avanti Polar lipids, Inc. (Alabaster, USA) |
| 66 | PG 16:0/18.1 | Phospholipids | >99% | Avanti Polar lipids, Inc. (Alabaster, USA) |
| 67 | PS 18:0/18:2 | Phospholipids | >99% | Avanti Polar lipids, Inc. (Alabaster, USA) |
| 68 | PE 16:0/18:1 | Phospholipids | >99% | Avanti Polar lipids, Inc. (Alabaster, USA) |
| 69 | PE 18:0/18:2 | Phospholipids | >99% | Avanti Polar lipids, Inc. (Alabaster, USA) |
| 70 | PC 16:0/18:0 | Phospholipids | >99% | Avanti Polar lipids, Inc. (Alabaster, USA) |
| 71 | PC 18:0/14:0 | Phospholipids | >99% | Avanti Polar lipids, Inc. (Alabaster, USA) |
| 72 | PC P-18:0/18:1 | Phospholipids | >99% | Avanti Polar lipids, Inc. (Alabaster, USA) |
| 73 | (^13^C-PC 16:0/16:0) | PL (IS) | 95% | Cambridge Isotope Laboratories, Inc. (Massachusetts, USA) |
| Reagent |  |  |  |  |
| 1 | Ultra-pure water (H_2_O) |  | Type I | Milli-Q purification system (Millipore, Bedford, MA) |
| 2 | Ammonium formate |  | >98% | Fisher Scientific (Morris Plains, NJ, USA) |
| 3 | formic acid |  | >99% | Fisher Scientific (Morris Plains, NJ, USA) |
| 4 | Acetonitrile |  | >99.9% | RCI Labscan (Bangkok, Thailand) |
| 5 | methanol |  | >99.8% | RCI Labscan (Bangkok, Thailand) |
| 6 | chloroform |  | >99.8% | RCI Labscan (Bangkok, Thailand) |
| 7 | Methyl tert-butyl ether |  | >99.5% | RCI Labscan (Bangkok, Thailand) |
| 8 | 2-propanol |  | >99.95% | RCI Labscan (Bangkok, Thailand) |

**Table S2. Selected quantifier and qualifier ions of amino acids by GC-MS/MS**

|  | Amino acids | RT | Quantifier | Qualifier |
| --- | --- | --- | --- | --- |
| 1 | Alanine | 7.645 | 260 | 158 |
| 2 | Glycine | 8.084 | 218 | 246 |
| 3 | Sarcosine | 8.679 | 260 | 232 |
| 4 | α-Aminobutyric acid | 8.791 | 172 | 246 |
| 5 | Valine | 9.632 | 186 | 288 |
| 6 | Leucine | 10.292 | 200 | 274 |
| 7 | Isoleucine | 10.731 | 200 | 302 |
| 8 | Norleucine (IS) | 10.949 | 200 | 274 |
| 9 | Proline | 11.174 | 184 | 258 |
| 10 | Methionine | 13.455 | 320 | 292 |
| 11 | Serine | 13.699 | 362 | 302 |
| 12 | Threonine | 14.044 | 303 | 404 |
| 13 | Phenylalanine | 15.106 | 302 | 336 |
| 14 | Aspartic acid | 16.042 | 316 | 418 |
| 15 | Cysteine | 16.808 | 304 | 406 |
| 16 | Glutamic acid | 17.462 | 330 | 272 |
| 17 | Ornithine | 17.528 | 286 | 184 |
| 18 | Asparagine | 17.691 | 302 | 417 |
| 19 | Lysine | 18.237 | 300 | 431 |
| 20 | Glutamine | 18.488 | 431 | 329 |
| 21 | Arginine | 18.872 | 442 | 340 |
| 22 | Histidine | 19.399 | 196 | 440 |
| 23 | Tyrosine | 19.663 | 302 | 466 |
| 24 | Tryptophan | 20.989 | 302 | 375 |

**Table S3 Selected quantifier and qualifier ions of organic acids by GC-MS/MS**

|  | Name | RT | Transition | Precursor Ion | Product Ion 1, (CE) | Product Ion 2, (CE) |
| --- | --- | --- | --- | --- | --- | --- |
| 1 | Formic acid | 3.5 | 103.0 -> 75.0 | 103 | 75, (7.5) | 47, (25) |
| 2 | Acetic acid | 4.0 | 117.0 -> 75.0 | 117 | 75, (10) | 47, (25) |
| 3 | Propionic acid | 4.7 | 131.0 -> 75.0 | 131 | 75, (12.5) | 47, (25) |
| 4 | Isobutyric acid | 5.14 | 145.0 -> 75.0 | 145 | 75, (10) | 47, (25) |
| 5 | Butyric acid | 5.25 | 145.0 -> 75.0 | 145 | 75, (10) | 47, (30) |
| 6 | Isovaleric acid | 5.58 | 159.0 -> 75.0 | 159 | 75, (10) | 57, (22.5) |
| 7 | Valeric acid | 5.88 | 159.0 -> 75.0 | 159 | 75, (12.5) | 57, (22.5) |
| 8 | Isocaproic acid | 6.28 | 173.0 -> 75.0 | 173 | 75, (2.5) | 81, (10) |
| 9 | Hexanoic acid | 6.52 | 173.0 -> 75.0 | 173 | 75, (2.5) | 131, (10) |
| 10 | Heptanoic acid | 7.38 | 187.0 -> 75.0 | 187 | 75, (2.5) | 131, (10) |
| 11 | Lactic acid | 8.4 | 261.0 -> 147.0 | 261 | 147, (7.5) | 43, (22.5) |
| 12 | Pyruvic acid | 8.65 | 259.0 -> 73.0 | 259 | 73, (17.5) | 147, (22.5) |
| 13 | Oxalic acid | 8.68 | 261.0 -> 73.0 | 261 | 73, (15) | 147, (22.5) |
| 14 | Oxaloacetic acid | 8.79 | 203.0 -> 73.0 | 203 | 73, (20) | 161, (5) |
| 15 | Malonic acid | 9.2 | 275.0 -> 73.0 | 275 | 73, (12.5) | 147, (30) |
| 16 | Methylmalonic acid | 9.28 | 289.0 -> 147.0 | 289 | 147, (7.5) | 73, (15) |
| 17 | Succinic acid | 9.58 | 289.0 -> 147.0 | 289 | 147, (10) | 73, (10) |
| 18 | Fumaric acid | 9.78 | 287.0 -> 73.0 | 287 | 73, (15) | 147, (22.5) |
| 19 | Malic acid | 10.73 | 287.0 -> 73.0 | 287 | 73, (17.5) | 147, (27.5) |
| 20 | Trans-cinnamic acid (ISTD) | 9.72 | 205.0 -> 131.0 | 205 | 131, (7.5) | 161, (12.5) |

**Table S4. Selected quantifier and qualifier ions of sugar and sugar alcohols by GC-TOFMS**

| Compound | RT | Quantifier | Quanlifier 1 | Quanlifier 2 | Quanlifier 3 | LOQ  (ug/L) | LOD  (ug/L) |
| --- | --- | --- | --- | --- | --- | --- | --- |
| Erythritol | 13.349 | 216.9048 | 75.0154 | 180.0217 | 146.8097 |  |  |
| D-(+)-Xylose | 15.006, 15.098 | 217.1075 | 307.1577 | 103.0574 | 147.0658 | 3.125 | 3.125 |
| D-(-)-Arabinose | 15.161 | 217.1074 | 307.1580 | 103.0574 | 147.0659 | 3.125 | 3.125 |
| D-(-)-Ribose | 15.333 | 217.1074 | 307.1578 | 103.0547 | 147.0659 | 3.125 | 3.125 |
| Xylitol | 15.681 | 217.1072 | 319.1577 | 205.1072 | 147.0660 | 3.125 | 3.125 |
| D-(+)-Arabitol | 15.813 | 217.1074 | 103.0574 | 205.1072 | 147.0660 | 6.25 | 3.125 |
| Adonitol | 15.873 | 147.0659 | 217.1075 | 205.1074 | 103.0574 | 10.00 | 6.25 |
| Fructose | 17.446,17.544 | 216.8481 | 73.0125 | 103.0329 | - |  |  |
| D-(+)-Mannose | 17.600, 17.762 | 147.0658 | 319.1578 | 205.1074 | 160.0790 | 10.00 | 10.00 |
| D-(+)-Galactose | 17.666, 17.881 | 147.0658 | 205.1072 | 160.0787 | 117.0366 | 10.00 | 6.25 |
| D-(+)-Glucose | 17.722, 17.911 | 147.0659 | 217.1074 | 205.1073 | 160.0790 | 10.00 | 6.25 |
| D-Mannitol | 18.050 | 147.0659 | 319.1577 | 217.1075 | 205.1074 | 10.00 | 6.25 |
| D-Sorbitol | 18.126 | 147.0658 | 217.1073 | 205.1073 | 157.0679 | 10.00 | 6.25 |
| Dulcitol | 18.169 | 147.0658 | 217.1073 | 205.1073 | 103.0573 | 10.00 | 6.25 |
| myo-Inositol | 19.568 | 147.0659 | 191.0916 | 305.1420 | 217.1073 | 10.00 | 6.25 |
| Sucrose | 24.187 | 361.1687 | 217.0713 | 437.2030 | 147.0657 | 10.00 | 6.25 |
| α-Lactose | 24.600, 24.719 | 204.0998 | 361.1684 | 217.1074 | 147.0657 | 25.00 | 10.00 |
| D-(+)-Maltose | 24.798, 25.169 | 204.0998 | 361.1683 | 217.1073 | 147.0658 | 25.00 | 10.00 |
| Maltitol | 25.686 | 204.0998 | 361.1687 | 217.1075 | 147.0655 | 10.00 | 6.25 |
| Myristic d-27 acid (ISTD) | 16.686 | 312.3936 | 132.0554 | 119.0491 | 76.0323, () | -, () | - |

**Table S5. LC-MS/MS MRM transitions and calibration curve linear range for phospholipid analysis.** All phospholipids were analyzed using the sample cone value of 20 V and the collision energy (CE) value of 10, except for the PG 16:0/18:1 where the CE was set at 25. The linearity (R²) was greater than 0.99 for all calibration curves. All phospholipid data were normalized by the internal standard,^13^C_40_-PC (16:0/16:0).

| **Compounds** | **Adduct** | **Parent ion (m/z)** | **Daughter ion (m/z)** | **Linear range (uM)** | |
| --- | --- | --- | --- | --- | --- |
|  |  |  |  | **LLoQ** | **ULoQ** |
| ^13^C_40_-PC (16:0/16:0) =IS | [M+H]+ | 774.70 | 189.11 | 0.0078 | 2.00 |
| PI 18:1/18:1 | [M+Na]+ | 885.54 | 603.53 | 0.0039 | 1.00 |
| PG 16:0/18:1 | [M+Na]+ | 771.51 | 194.99 | 0.0020 | 0.25 |
| PS (18:0/18:2) | [M+H]+ | 788.54 | 603.5 | 0.0020 | 0.50 |
| PC (18:0/14:0) | [M+H]+ | 734.57 | 184.07 | 0.0313 | 4.00 |
| PE (16:0/18:1) | [M+H]+ | 718.54 | 577.52 | 0.0039 | 1.00 |
| PE (18:0/18:2) | [M+H]+ | 744.55 | 603.53 | 0.0313 | 4.00 |
| PC (18:0/16:0) | [M+H]+ | 726.60 | 124.99 | 0.0078 | 1.00 |
| PC (18:0/18:1) | [M+H]+ | 772.62 | 184.07 | 0.0039 | 1.00 |

**Table S6. Performance of plasma metabolites to differentiate between NSCLC patients and healthy subjects.**

| No | Metabolite | AUC | Accuracy | Sensitivity | Specificity |
| --- | --- | --- | --- | --- | --- |
| 1 | Glycine | 1.000 | 1.000 | 1.000 | 1.000 |
| 2 | Propionic acid | 1.000 | 1.000 | 1.000 | 1.000 |
| 3 | Maltose | 1.000 | 1.000 | 1.000 | 1.000 |
| 4 | Tryptophan | 1.000 | 0.985 | 1.000 | 0.983 |
| 5 | Pyruvic acid | 0.998 | 0.985 | 1.000 | 0.982 |
| 6 | Inositol | 1.000 | 0.985 | 1.000 | 0.982 |
| 7 | Sarcosine | 0.984 | 0.971 | 0.917 | 0.983 |
| 8 | Arginine/citrulline/ornithine | 0.983 | 0.957 | 0.917 | 0.964 |
| 9 | Oxalic acid | 0.981 | 0.956 | 0.792 | 0.982 |
| 10 | Histidine | 0.898 | 0.943 | 0.917 | 0.949 |

AUC, area under the receiver operating characteristic curve

Table S7 **Performance of plasma metabolites to differentiate between NSCLC patients with EGFR mutation and without EGFR mutation.**

| Metabolite | AUC | Accuracy | Sensitivity | Specificity |
| --- | --- | --- | --- | --- |
| Valine | 0.782 | 0.757 | 0.830 | 0.692 |
| Isoleucine | 0.743 | 0.707 | 0.723 | 0.696 |
| Leucine | 0.785 | 0.706 | 0.723 | 0.692 |
| PC 16:0/18:0 | 0.721 | 0.705 | 0.683 | 0.723 |
| Sucrose | 0.641 | 0.692 | 0.763 | 0.607 |
| PS 18:0/18:2 | 0.678 | 0.642 | 0.625 | 0.665 |
| Oxalic acid | 0.606 | 0.638 | 0.522 | 0.763 |
| Fumaric acid | 0.610 | 0.620 | 0.688 | 0.554 |
| Tryptophan | 0.766 | 0.619 | 0.696 | 0.558 |
| Formic acid | 0.675 | 0.618 | 0.688 | 0.545 |

AUC, area under the receiver operating characteristic curve

Table S8. Summary of quantified metabolites and results of multiple regression analysis (Table S8.xlsx)

Table S9. Performance of plasma metabolites to differentiate between treatment-naïve NSCLC patients with EGFR mutation and TKI-resistant group

| Metabolite | AUC | Accuracy | Sensitivity | Specificity |
| --- | --- | --- | --- | --- |
| Tryptophan | 0.803 | 0.822 | 0.902 | 0.625 |
| Oxalic acid | 0.759 | 0.822 | 0.969 | 0.417 |
| Sarcosine | 0.648 | 0.797 | 1.000 | 0.208 |
| Sucrose | 0.752 | 0.794 | 0.933 | 0.375 |
| Succinic acid | 0.817 | 0.792 | 0.897 | 0.500 |
| Valine | 0.693 | 0.772 | 0.933 | 0.333 |
| Aspartic acid | 0.772 | 0.769 | 0.929 | 0.292 |
| Propionic acid | 0.721 | 0.769 | 0.964 | 0.208 |
| Glutamic acid | 0.686 | 0.769 | 0.964 | 0.167 |
| Formic acid | 0.659 | 0.769 | 1.000 | 0.083 |

AUC, area under the receiver operating characteristic curve

**Table S10. Calibration parameters of targeted metabolites analyzed by GC-MS**

| Compound | Calibration curve range (ng/injection) | R^2^ | LOQ  (ng/injection) | LOD  (ng/injection) | %RSD  (n=3) |
| --- | --- | --- | --- | --- | --- |
| Alanine | 0.9766 - 1000 | 0.9977 | 0.9766 | 0.9766 | 3.52 |
| Glycine | 0.9766 - 1000 | 0.9913 | 0.9766 | 0.9766 | 8.99 |
| Sarcosine | 0.9766 - 1000 | 0.9987 | 0.9766 | 0.9766 | 0.32 |
| α-Aminobutyric acid | 0.9766 - 1000 | 0.9988 | 0.9766 | 0.9766 | 0.87 |
| Valine | 0.9766 - 1000 | 0.9983 | 0.9766 | 0.9766 | 1.27 |
| Leucine | 0.9766 - 1000 | 0.9985 | 0.9766 | 0.9766 | 1.44 |
| Isoleucine | 0.9766 - 1000 | 0.9979 | 0.9766 | 0.9766 | 0.66 |
| Proline | 0.9766 - 1000 | 0.9925 | 0.9766 | 0.9766 | 3.12 |
| Methionine | 0.9766 - 1000 | 0.9979 | 0.9766 | 0.9766 | 5.42 |
| Serine | 0.9766 - 1000 | 0.9976 | 0.9766 | 0.9766 | 6.97 |
| Threonine | 0.9766 - 1000 | 0.9984 | 0.9766 | 0.9766 | 9.79 |
| Phenylalanine | 0.9766 - 1000 | 0.9995 | 0.9766 | 0.9766 | 4.95 |
| Aspartic acid | 0.9766 - 1000 | 0.9981 | 0.9766 | 0.9766 | 5.82 |
| Cysteine | 0.9766 - 250 | 0.9838 | 0.9766 | 0.9766 | 4.67 |
| Glutamic acid | 0.9766 - 1000 | 0.9945 | 0.9766 | 0.9766 | 1.30 |
| Ornithine | 0.9766 - 250 | 0.9971 | 0.9766 | 0.9766 | 8.93 |
| Asparagine | 0.9766 - 500 | 0.9924 | 0.9766 | 0.9766 | 2.23 |
| Lysine | 0.9766 - 250 | 0.9961 | 0.9766 | 0.9766 | 9.40 |
| Glutamine | 62.500 - 250 | 0.9951 | 62.500 | 31.250 | 7.28 |
| Arginine | 0.9766 - 1000 | 0.9931 | 0.9766 | 0.9766 | 1.49 |
| Histidine | 62.500 - 250 | 0.9981 | 62.500 | 31.250 | 6.85 |
| Tyrosine | 0.9766 - 1000 | 0.9931 | 0.9766 | 0.9766 | 6.74 |
| Tryptophan | 15.6250 - 500 | 0.9961 | 15.625 | 15.625 | 3.10 |
| Cystine | 0.9766 - 1000 | 0.9951 | 0.9766 | 0.9766 | 2.84 |
| Formic acid | 0.3382-86.5801 | 0.9984 | 0.0382 | 0.0382 | 0.24 |
| Formic acid | 0.3382-86.5801 | 0.9984 | 0.0382 | 0.0382 | 0.24 |
| Acetic acid | 0.3382-86.5801 | 0.9975 | 0.0382 | 0.0382 | 8.18 |
| Propionic acid | 0.3382-86.5801 | 0.9927 | 0.0382 | 0.0382 | 0.47 |
| Isobutyric acid | 0.3382-86.5801 | 0.9993 | 0.0382 | 0.0382 | 9.20 |
| Butyric acid | 0.3382-86.5801 | 0.9997 | 0.0382 | 0.0382 | 9.03 |
| Isovaleric acid | 0.3382-86.5801 | 0.9996 | 0.0382 | 0.0382 | 4.52 |
| Valeric acid | 0.3382-86.5801 | 0.9993 | 0.0382 | 0.0382 | 6.20 |
| Isocaproic acid | 0.3382-86.5801 | 0.9992 | 0.0382 | 0.0382 | 5.05 |
| Hexanoic acid | 0.3382-86.5801 | 0.9998 | 0.0382 | 0.0382 | 6.05 |
| Heptanoic acid | 0.3382-86.5801 | 0.9997 | 0.0382 | 0.0382 | 3.36 |
| Lactic acid | 27.0563-432.9004 | 0.9981 | 0.0382 | 0.8455 | 3.08 |
| Pyruvic acid | 27.0563-432.9004 | 0.9989 | 0.0382 | 0.0382 | 6.36 |
| Oxalic acid | 0.3382-86.5801 | 0.9986 | 0.0382 | 0.0382 | 3.18 |
| Oxaloacetic acid | 0.3382-43.2900 | 0.9987 | 0.3382 | 0.3382 | 5.47 |
| Malonic acid | 0.3382-86.5801 | 0.9994 | 0.0382 | 0.0382 | 2.42 |
| Metmaloic acid | 0.3382-86.5801 | 0.9997 | 0.0382 | 0.0382 | 3.05 |
| Succinic acid | 0.3382-86.5801 | 0.9997 | 0.0382 | 0.0382 | 0.86 |
| Fumaric acid | 0.3382-86.5801 | 0.9986 | 0.0382 | 0.0382 | 0.75 |
| Malic acid | 0.0845-216.4502 | 0.9999 | 0.0382 | 0.0382 | 7.44 |
| Meso-Erythritol | 1.5630 - 100 | 0.9991 | 1.5630 | 0.0390 | 0.54 |
| D-(+)-Xylose | 1.5630 - 100 | 0.9989 | 1.5630 | 0.0390 | 0.29 |
| D-(-)-Arabinose | 1.5630 - 100 | 0.9944 | 1.5630 | 0.0390 | 0.71 |
| D-(-)-Ribose | 1.5630 - 200 | 0.9997 | 1.5630 | 0.0390 | 3.93 |
| Xylitol | 1.5630 - 100 | 0.9991 | 1.5630 | 0.0390 | 0.84 |
| D-(+)-Arabitol | 1.5630 - 100 | 0.9990 | 1.5630 | 0.0390 | 0.78 |
| Adonitol | 1.5630 - 100 | 0.9993 | 1.5630 | 0.0390 | 0.95 |
| Fructose | 3.1250 - 100 | 0.9939 | 3.1250 | 0.0390 | 2.23 |
| D-(+)-Mannose | 1.5630 - 100 | 0.9989 | 1.5630 | 0.0390 | 0.44 |
| D-(+)-Galactose | 1.5630 - 100 | 0.9982 | 1.5630 | 0.0390 | 0.06 |
| D-(+)-Glucose | 1.5630 - 100 | 0.9987 | 1.5630 | 0.0390 | 0.15 |
| D-Mannitol | 6.2500 - 200 | 0.9984 | 6.2500 | 0.0390 | 7.50 |
| D-Sorbitol | 6.2500 - 100 | 0.9974 | 6.2500 | 0.0390 | 0.38 |
| Dulcitol | 6.2500 - 100 | 0.9975 | 6.2500 | 0.0390 | 1.29 |
| myo-Inositol | 1.5630 - 500 | 0.9968 | 1.5630 | 0.0390 | 0.25 |
| Sucrose | 3.1250 - 100 | 0.9999 | 3.1250 | 0.0390 | 3.31 |
| α-Lactose | 6.2500 - 100 | 0.9993 | 6.2500 | 0.0390 | 0.52 |
| D-(+)-Maltose | 3.1250 - 100 | 0.9993 | 3.1250 | 0.0390 | 1.18 |
| Maltitol | 6.2500 - 100 | 0.9989 | 6.2500 | 0.0390 | 2.05 |
